# Awareness, Perception, Utilization, and Challenges of Ayushman Bharat-Pradhan Mantri Jan Arogya Yojana Among Peri-Urban Residents of Kanpur, Uttar Pradesh: A Community-Based Cross-Sectional Study

**DOI:** 10.7759/cureus.117489

**Published:** 2026-10-05

**Authors:** Saket Shekhar, Abhishek Singh, Poonam Kushwaha, Anubhuti Sharma, Tarun Chauhan, Bijit Biswas, Shivam Rai

**Affiliations:** 1 Community Medicine, Employees' State Insurance Corporation (ESIC) Medical College Hospital, Patna, IND; 2 Community Medicine, Rama Medical College Hospital and Research Centre, Kanpur, IND; 3 Community Medicine, Shree Narayan Medical Institute and Hospital, Saharsa, IND; 4 Community and Family Medicine, All India Institute of Medical Sciences, Deoghar, IND

**Keywords:** access to healthcare, ayushman bharat, health insurance, health service utilization, india, pm-jay

## Abstract

Background

Ayushman Bharat-Pradhan Mantri Jan Arogya Yojana (AB-PMJAY) is the world's largest publicly funded health assurance scheme aimed at reducing catastrophic health expenditure and improving access to secondary and tertiary healthcare services among economically vulnerable populations. Despite its wide coverage, awareness and utilization of the scheme vary across regions of India. This study aimed to assess the awareness, perceptions, utilization, and challenges related to AB-PMJAY among peri-urban residents of Kanpur and to identify factors associated with awareness of the scheme.

Methods

A community-based cross-sectional study was conducted among 214 adults residing in the peri-urban areas of Mandhana and Chaubeypur, Kanpur, Uttar Pradesh. A multistage random sampling technique was used for participant selection. Data were collected using a pretested semi-structured questionnaire through face-to-face interviews. Awareness regarding PM-JAY was assessed using a composite awareness score. Data were analyzed using jamovi Version 2.3.18 (The jamovi project (2023); retrieved from https://www.jamovi.org). Descriptive statistics were expressed as frequencies and percentages, and the chi-squared test was applied to determine associations. A p-value of <0.05 was considered statistically significant.

Results

Among the 214 participants, 127 (59.3%) demonstrated good awareness regarding AB-PMJAY. The highest awareness was observed regarding ₹5 lakhs annual family coverage (75.7%), whereas awareness regarding post-discharge benefits was very low (3.7%). Among the eligible participants, 77.3% had obtained PM-JAY cards; however, only 45.1% had utilized PM-JAY services. Out-of-pocket expenditure despite PM-JAY utilization was reported by 39.1% of PM-JAY users, and 26.1% experienced delays in healthcare services. Educational status and possession of a ration card were significantly associated with awareness of PM-JAY. Most participants expressed a positive perception of the improvement in healthcare access under PM-JAY.

Conclusion

The study found moderate awareness and good enrolment regarding AB-PMJAY among peri-urban residents; however, actual utilization remained suboptimal. Significant gaps existed in the awareness of specific scheme benefits, particularly post-discharge benefits and family coverage provisions. Strengthening community-based awareness activities and addressing implementation-related barriers such as out-of-pocket expenditure and service delays may improve effective utilization of AB-PMJAY services. Education status and possession of ration cards were significantly associated with awareness.

## Introduction

Out-of-pocket (OOP) expenditure refers to the cost of healthcare that individuals pay at the time of treatment through user fees, co-payments, and direct payments for medicines [1]. According to the World Health Organization in 2021, OOP health expenditure comprises 49% of the current health expenditure in India. The increasing burden of catastrophic health expenditure (CHE) poses a significant threat to individuals and households in India, where OOP constitutes a substantial portion of healthcare financing [2]. A recent meta-analysis indicated that the estimated incidence of CHE at a 10% threshold was 0.30 (0.25-0.35), indicating a significant prevalence of financial hardship due to health expenses [3]. To address this problem, the United Nations developed the Sustainable Development Goal (SDG) Target 3.8, which aims to achieve universal health coverage. The coverage of essential health services and prevention of catastrophic health spending are the two components of universal health coverage [4].

To meet SDG health-related goals, the Indian government approved a new National Health Policy in 2017 [5]. Ayushman Bharat is a flagship scheme to meet the target of the National Health Policy launched in 2018 [6]. It included two major initiatives, namely, the creation of health and wellness centers (HWCs) and Pradhan Mantri Jan Arogya Yojana (PM-JAY) [7]. Ayushman Bharat-Pradhan Mantri Jan Arogya Yojana (AB-PMJAY) is the largest health assurance scheme in the world. It aims to provide a health cover of ₹5 lakhs per family per year for secondary and tertiary care hospitalization to over 12 crores of poor and vulnerable families (approximately 55 crore beneficiaries) that form the bottom 40% of the Indian population. The households included are based on the deprivation and occupational criteria of the Socio-Economic Caste Census (SECC) 2011 for rural and urban areas, respectively [8]. By August 2020, around 12.5 crore E-cards and 1.09 crore hospital admissions have been done under this scheme. Various studies from different parts of India suggest very low to high awareness and utilization of the PM-JAY scheme [9-11]. Evidence regarding awareness, perception, and utilization of PM-JAY among peri-urban populations of northern India remains limited. Therefore, this study was conducted to assess the awareness, perception, utilization, and determinants of awareness regarding AB-PMJAY among peri-urban residents of Kanpur, Uttar Pradesh.

## Materials and methods

Study setting and design

This was a community-based cross-sectional study conducted in a peri-urban location in Kanpur. The peri-urban areas of Mandhana and Chaubeypur in Kanpur are characterized by mixed rural-urban socioeconomic conditions and dependence on both public and private healthcare facilities. Healthcare services in these areas are delivered through primary health centers, community health centers, private clinics, empanelled hospitals under PM-JAY, and tertiary care institutions located within Kanpur city. Residents commonly access healthcare services from nearby government facilities and private healthcare providers due to perceived better accessibility and quality of care. The study was conducted from March 2024 to August 2024.

Study population

The study population comprised adult residents aged 18 years and above residing in the peri-urban areas of Mandhana and Chaubeypur in Kanpur. The study included heads of households or, in their absence, the eldest available adult member of the household who had been residing in the area for at least six months and was willing to participate in the study. Individuals who were severely ill, cognitively impaired, or unwilling to provide informed consent were excluded from the study.

Sample size and sampling strategy

The sample size was calculated using the formula $\mathrm{n}=4\mathrm{pq}/\mathrm{L}^{2}$. Assuming awareness of PM-JAY as 50% in the absence of prior local data and an allowable error of 7%, the minimum required sample size was 204 participants. However, a total of 214 participants were included to adjust for non-response. A multistage random sampling technique was employed. In the first stage, four villages were selected using simple random sampling from the rural field practice area. In the second stage, households within each selected village were selected using systematic random sampling. The sampling interval was calculated based on the total number of households and required sample size. The sampling interval was approximately every fifth household in the village. The head of the household was selected for the interview. If the head of the household was unavailable, the eldest available adult member of the household was included in the study.

Instrument description

A semi-structured, closed-ended questionnaire was prepared (Appendices). The questionnaire was translated from English to Hindi for better comprehension among the study participants. Data were collected through face-to-face interviews. The questionnaire comprised five sections. The first section consisted of the sociodemographic characteristics of the study participants, including age group, gender, religion, occupation, type of family, caste, and possession of a ration card. The second section consisted of the healthcare-seeking behavior of the study participants. The third section was about awareness of PM-JAY and its various provisions, such as card portability, free coverage, coverage amount, family floater system, and post-discharge benefits. The fourth section consisted of questions regarding their eligibility for PM-JAY and their perceptions of it. The fourth section consisted of questions regarding participants' self-reported eligibility for PM-JAY and their perceptions of the scheme. Participants were asked whether they considered themselves eligible for an AB-PMJAY card, with responses recorded as "Yes", "No", or "Don't know". Eligibility was not independently verified against official PM-JAY records. The fifth section included questions regarding the utilization of the PM-JAY card and challenges encountered during its use. Awareness of AB-PMJAY was assessed using a study-specific composite awareness score derived from predefined PM-JAY awareness-related questions in the questionnaire. Each correct response was assigned a score of 1, while incorrect or "Don't know" responses were assigned a score of 0. Participants scoring ≥50% of the total obtainable score were categorized as having good awareness, whereas those scoring <50% were categorized as having poor awareness. The questionnaire was pilot-tested in the Kalyanpur area among 15 residents. Pilot data were not included in this study.

Data management and analysis

Data analysis was performed using jamovi Version 2.3.18 (The jamovi project (2023); retrieved from https://www.jamovi.org). Prior to analysis, the collected data were checked for completeness, consistency, and accuracy. Participants with incomplete or missing responses were excluded from the analysis. Data were also checked for duplicate entries, coding errors, and inconsistencies in categorical variables. Qualitative variables are presented as frequencies and percentages. The association between categorical variables and awareness of PM-JAY was assessed using unadjusted bivariate analysis with the chi-squared test. A p-value of <0.05 was considered statistically significant. Certain variables were regrouped for analytical purposes to ensure adequate cell frequencies and meaningful interpretation during the chi-squared analysis. Age was dichotomized as <30 and ≥30 years, and education was categorized as illiterate, primary, and secondary and above, while caste categories were grouped as general and other backward class (OBC)/scheduled caste (SC)/scheduled tribe (ST).

Ethical considerations

Ethical clearance for the study was obtained from the Institutional Medical Ethics Committee of Rama Medical College Hospital and Research Centre before the commencement of the study (approval number: RMCHRC/Ethics/2024/3371-B; approval date: 05.03.2024). Written informed consent was obtained from all the study participants before data collection. Confidentiality and anonymity of the participants' information were maintained throughout the study.

## Results

A total of 214 participants were included in this study. The sociodemographic characteristics of the study participants are presented in Table 1. The majority of participants were aged under 30 years (78, 36.4%), followed by those aged 30-45 years (61, 28.5%). Nearly two-thirds of the participants were men. Most participants were Hindus (176, 82.2%), and over half belonged to the general caste category (110, 51.4%). In terms of education, 67 (31.3%) had attained higher secondary education and above, while 49 (22.9%) were illiterate. A large proportion (168, 78.5%) of the respondents possessed ration cards.

**Table 1:** Sociodemographic characteristics of the study participants (n=214)

Variable	Category	Frequency (n)	Percentage (%)
Age group (years)	<30	78	36.4
	30-45	61	28.5
	46-60	49	22.9
	≥61	26	12.2
Gender	Male	146	68.2
	Female	67	31.3
	Others	1	0.5
Religion	Hindu	176	82.2
	Muslim	34	15.9
	Others	4	1.9
Caste	General	110	51.4
	Other backward class (OBC)	72	33.6
	Scheduled caste (SC)/scheduled tribe (ST)	32	15
Education status	Illiterate	49	22.9
	Primary	40	18.7
	Secondary	58	27.1
	Higher secondary and above	67	31.3
Occupation	Unskilled	42	19.6
	Semi-skilled	38	17.8
	Skilled	56	26.2
	Others	78	36.4
Type of family	Nuclear	102	47.7
	Joint	89	41.6
	Others	23	10.7
Ration card	Yes	168	78.5
	No	46	21.5

With respect to healthcare-seeking behavior, nearly two-thirds of the study participants reported easy access to healthcare services, while 48 (22.4%) and 20 (9.4%) reported moderate and difficult access, respectively. Healthcare decisions were predominantly made by family members (108, 50.5%), followed by self (95, 44.4%). Regarding the frequency of healthcare visits, 78 (36.4%) of the participants reported visiting healthcare facilities monthly. A clear preference for private healthcare was observed, with 142 (66.4%) opting for private outpatient services and 125 (58.4%) opting for private inpatient care (Table 2).

**Table 2:** Healthcare-seeking characteristics of the study participants (n=214)

Variable	Category	Frequency (n)	Percentage (%)
Access to healthcare services	Easy	146	68.2
	Moderate	48	22.4
	Difficult	20	9.4
Decision-maker for healthcare	Self	95	44.4
	Family member	108	50.5
	Others (friends/relatives)	11	5.1
Frequency of healthcare visits	Very rarely	28	13.1
	Once a year	33	15.4
	Every 6 months	54	25.2
	Every month	78	36.4
	Every week	21	9.9
Preferred outpatient facility	Government	72	33.6
	Private	142	66.4
Preferred inpatient facility	Government	89	41.6
	Private	125	58.4

Awareness of PM-JAY

Table 3 summarizes the overall awareness of PM-JAY. Of the 214 participants, 127 (59.3%) demonstrated good awareness, while 87 (40.7%) had poor awareness. A detailed assessment of awareness of specific PM-JAY components is presented in Table 3. Among all participants, the highest awareness was observed for the financial benefit of up to ₹5 lakhs annual coverage per family (162, 75.7%). Awareness that the PM-JAY card is provided free of cost was reported by 111 (51.9%), while 102 (47.7%) were aware that the scheme covers hospitalization expenses. Awareness of cashless treatment across India (card portability) was reported by 104 (48.6%) of the participants. However, awareness of the scheme's more detailed aspects was comparatively low. Only 100 (46.7%) were aware that eligibility is based on socioeconomic criteria, and 94 (43.9%) knew that the scheme covers pre-existing illnesses. Only one-third of the participants were aware that the benefits extended to the entire family. Notably, awareness of post-discharge benefits was extremely low, with only eight (3.7%) of respondents reporting knowledge of this component. When awareness was assessed among eligible participants (n=132), higher levels of awareness were consistently observed across all components (Table 3). Awareness of ₹5 lakh coverage was 115 (87.1%), while 90 (68.2%) knew that the card was free of cost, and 86 (65.2%) were aware of hospitalization coverage. Awareness of cashless treatment (82, 62.1%) and eligibility criteria (74, 56.1%) was also higher among eligible individuals. Despite this, gaps persisted even among eligible participants, particularly in post-discharge benefits (5, 3.8%) and family coverage (50, 37.9%).

**Table 3:** Awareness regarding various components of AB-PMJAY among total and eligible participants AB-PMJAY: Ayushman Bharat-Pradhan Mantri Jan Arogya Yojana

Awareness component	Among total participants (n=214) n (%)	Among eligible participants (n=132) n (%)
Covers hospitalization expenses	102 (47.7)	86 (65.2)
PM-JAY card is free of cost	111 (51.9)	90 (68.2)
Provides ₹5 lakh coverage per family per year	162 (75.7)	115 (87.1)
Covers all existing illnesses	94 (43.9)	72 (54.5)
Provides post-discharge benefits	8 (3.7)	5 (3.8)
Benefits available to the entire family	68 (31.8)	50 (37.9)
Eligibility based on socioeconomic criteria	100 (46.7)	74 (56.1)
Offers cashless treatment across India	104 (48.6)	82 (62.1)

Eligibility, utilization, and challenges

The eligibility, enrolment, and utilization patterns of PM-JAY are presented in Table 4. Of the total participants, 132 (61.7%) reported being eligible for the scheme. Among these participants, 102/132 (77.3%) had obtained a PM-JAY card. However, only 46/102 (45.1%) of those possessing a PM-JAY card had utilized PM-JAY services. A total of 18 (39.1%) participants incurred OOP expenditures despite being covered under the scheme, while 12 (26.1%) reported delays in receiving healthcare services.

**Table 4:** Eligibility, enrolment, and utilization of PM-JAY card among the study participants PM-JAY: Pradhan Mantri Jan Arogya Yojana

Variable	Category	Frequency (n)	Percentage (%)
Eligibility for PM-JAY (n=214)	Yes	132	61.7
	No	82	38.3
Obtained PM-JAY card (n=132)	Yes	102	77.3
	No	30	22.7
Utilized PM-JAY services (n=102)	Yes	46	45.1
	No	56	54.9
Out-of-pocket expenditure despite PM-JAY use (n=46)	Yes	18	39.1
	No	28	60.9
Delay in receiving services (n=46)	Yes	12	26.1
	No	34	73.9

Perception regarding PM-JAY

Figure 1 illustrates the participants' perceptions of improvements in healthcare access under PM-JAY. Most participants expressed a positive perception, with most respondents either agreeing or strongly agreeing that PM-JAY improved access to healthcare services. A higher proportion of eligible participants reported strong agreement than the overall study population, indicating that scheme beneficiaries perceive greater benefits.

**Figure 1:**
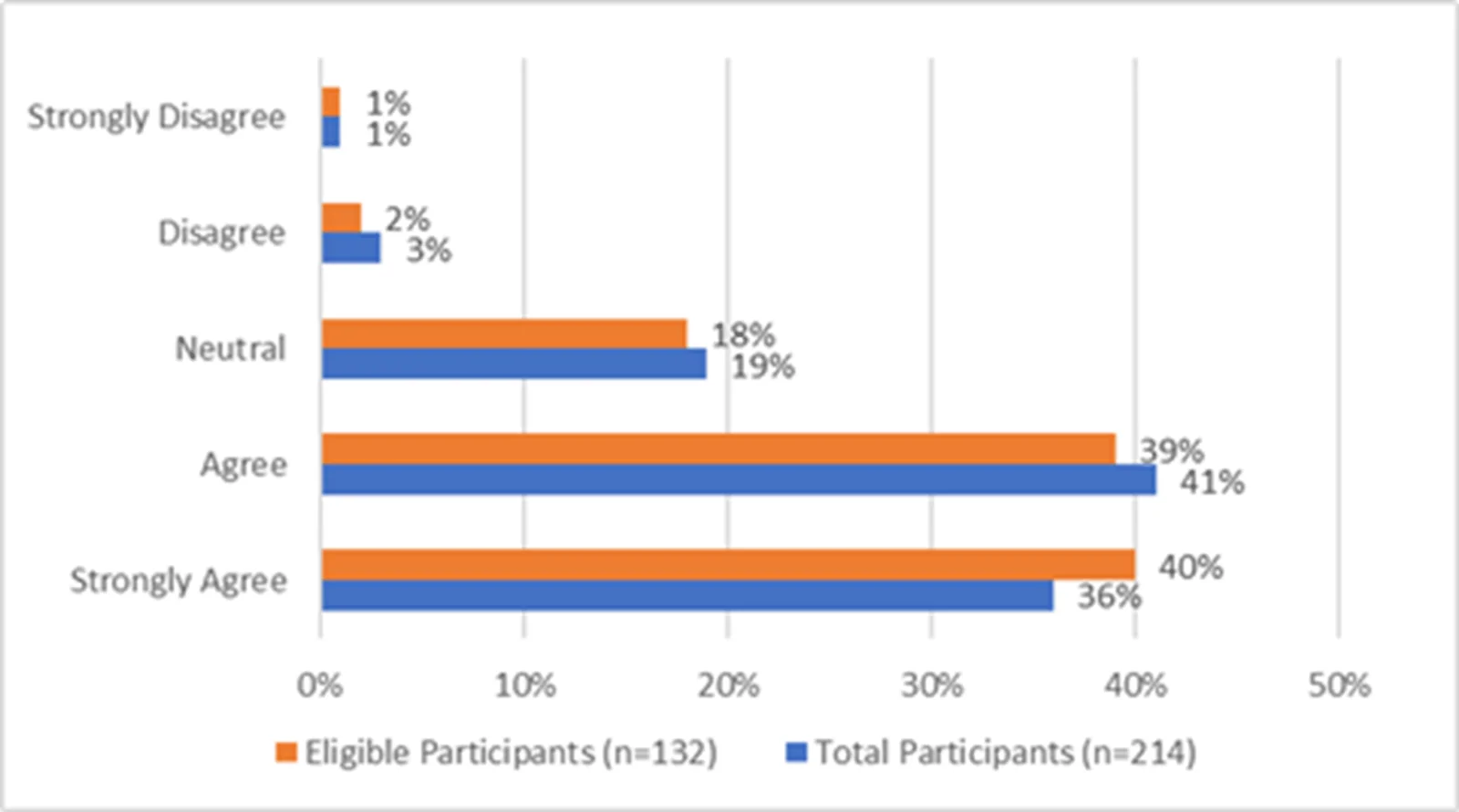
Perception of PM-JAY regarding improvement in healthcare access among total and eligible participants PM-JAY: Pradhan Mantri Jan Arogya Yojana

Determinants of awareness

The factors associated with the awareness of PM-JAY among the study participants are presented in Table 5. The educational status of the study participants was significantly associated with awareness. Participants with secondary and above education demonstrated substantially higher levels of good awareness (91, 72.8%) than those who were illiterate (16, 32.7%). Possession of a ration card was also significantly associated with awareness, with 110 (65.5%) of ration card holders having good awareness compared to 17 (37%) among those without a card. In contrast, age group, sex, and caste were not significantly associated with awareness.

**Table 5:** Association of sociodemographic variables with awareness regarding AB-PMJAY among study participants *Age categories dichotomized for analysis (<30 years vs. ≥30 years). #Education categories were grouped for analytical purposes. †One participant in the "Others" gender category; included for completeness but not considered in the χ² test due to the small cell size. AB-PMJAY: Ayushman Bharat-Pradhan Mantri Jan Arogya Yojana; OBC: other backward class; SC: scheduled caste; ST: scheduled tribe

Variable	Category	Poor awareness (n=87) n (%)	Good awareness (n=127) n (%)	χ² (df)	P-value
Age group*	<30 years	35 (44.9)	43 (55.1)	0.905 (1)	0.341
	≥30 years	52 (38.2)	84 (61.8)		
Gender	Male	60 (41.1)	86 (58.9)	0.01 (1)	0.910
	Female	27 (40.3)	40 (59.7)		
	Others†	0 (0)	1 (100)		
Education status^#^	Illiterate	33 (67.3)	16 (32.7)	17.03 (2)	<0.001
	Primary	20 (50)	20 (50)		
	Secondary and above	34 (27.2)	91 (72.8)		
Ration card	Yes	58 (34.5)	110 (65.5)	12.17 (1)	0.001
	No	29 (63)	17 (37)		
Caste	General	42 (38.2)	68 (61.8)	0.24 (1)	0.623
	OBC/SC/ST	45 (43.3)	59 (56.7)		

## Discussion

The present study assessed the awareness, perception, and utilization of AB-PMJAY among peri-urban residents of Kanpur District, Uttar Pradesh, India. Data were collected from 214 randomly selected residents. The findings revealed moderate awareness (59.3%), high enrolment among eligible participants (77.3%), but substantially lower utilization (45.1%), alongside persistent implementation challenges, including OOP expenditure and service delays.

The observed awareness level of 59.3% in the present study was considerably higher than that reported in other parts of Uttar Pradesh. Similar findings have been reported in another study in Eastern India in the rural area of Patna, Bihar [12]. In another study from rural Meerut, Uttar Pradesh, awareness was approximately 72.6%. A large proportion of eligible households in six Indian states reported 62% awareness of PM-JAY [11]. Another study in Chhattisgarh, conducted among eligible beneficiaries, reveals a very high level of awareness (90%) [13]. In another study in rural Puducherry, among below-poverty-line households, awareness was found to be 71.7%. Another study in Gujarat in three districts reported that 24% and 47.8% of the respondents had high and moderate levels of awareness, respectively. However, another study from Gautam Budha Nagar, Uttar Pradesh, compared awareness among rural and urban residents and reported that 8.9% and 18.8% of beneficiaries in urban and rural areas, respectively, were aware of the benefits of the scheme [14]. Taken together, these findings suggest that awareness of PM-JAY remains moderate across many regions of India.

In our study, we also inquired in depth regarding various aspects of PM-JAY, such as eligibility criteria, coverage amount, family floater, card portability across India, no premium to be paid, and post-discharge benefits. This was done to assess the areas of PM-JAY where a lack of information persists. Our study found that post-discharge benefits are one of the least informed areas of PM-JAY. Another common lack of information was about the family floater system in PM-JAY. A similar pattern was observed in both the general study population and PM-JAY-eligible beneficiaries. However, most studies have not evaluated this aspect. However, similar findings of very low awareness of post-discharge benefits have been reported by a few studies [12,15]. Post-discharge benefits in PM-JAY include free medicine, diagnostics, consultation, and treatment of complications up to 15 days after discharge [16,17]. This is one key provision that must decrease OOP expenditure and further increase the utilization of AB-PMJAY.

We assessed perceptions of PM-JAY among the general and eligible populations. We inquired about improvements in healthcare access after PM-JAY. The assessment was performed using a 5-point Likert scale. Most of the responses were positive and very positive [17,18]. A mixed-methods study reported similar findings. However, this study also reported the need for communication skills and empathetic and coordinated care by PM-JAY implementers and healthcare providers.

Our study reported that enrolment among PM-JAY-eligible patients was 77.3%. However, only half of them utilized PM-JAY. Our findings of lower utilization despite high enrolment were consistent with most previous studies [12,14,19]. A rural Bihar study found that only 1.3% of the eligible population utilized the scheme. In Gujarat, almost one in two PM-JAY-eligible individuals utilized the scheme. In rural Ayodhya, one in two had utilized the facility [20]. The difference in the utilization rate might be due to a difference in the denominator. Some studies reported data on eligible individuals, while others reported data on enrolled individuals. The utilization rate might have improved over time since seven years have passed since the commencement of the scheme. More private and government hospitals have been empanelled into the scheme, and the number of packages offered has also improved with time [21,22].

The challenges faced by PM-JAY card users in our study were mainly two. The first was substantial OOP expenditure even with the scheme. Approximately two-fifths of PM-JAY users reported OOP expenditure despite utilizing the scheme. Similar findings have been reported in previous studies. A hospital-based study in Gujarat and Madhya Pradesh also reported that OOP expenditure is higher in private hospitals than in government hospitals [18]. A mixed-methods study also reported that 29% incurred pre-hospitalization OOP expenditure for travel, consultation, tests, medicine, and follow-up visits [17]. Another reason cited for OOP expenditure was paying the difference between the provider-preferred drug or implant and what PM-JAY covers [23]. The second major challenge was delays in receiving services, reported by 26% of PM-JAY users. Our findings about service delays with PM-JAY were consistent with many previous studies [17,23,24]. Pre-authorization approval sometimes took up to 24 hours to several days, which delays the start of the treatment. Solving these issues will likely increase utilization and patient satisfaction among PM-JAY beneficiaries.

Educational status and possession of a ration card were significantly associated with awareness of PM-JAY, highlighting the importance of socioeconomic and informational determinants in improving scheme awareness. Participants with higher educational attainment demonstrated substantially better awareness than illiterate participants. The findings of the present study emphasize the need to strengthen community-based information, education, and communication (IEC) activities, particularly among socioeconomically vulnerable and less educated populations. Focused awareness campaigns through frontline health workers, local governance systems, and mass media may improve understanding of the benefits and utilization process of PM-JAY. Furthermore, addressing implementation barriers, such as OOP expenditure and service delays, is essential to enhance beneficiary trust, improve utilization, and achieve the intended goal of financial risk protection under AB-PMJAY.

This study has certain limitations. Being cross-sectional in nature, causal relationships between variables cannot be established. Information was collected through self-reports and may therefore be subject to recall bias. In addition, the study was conducted in selected peri-urban areas of Kanpur, which may limit the generalizability of the findings to other settings in India. Although multistage sampling was employed, clustering at the village level was not specifically accounted for in the statistical analysis and may have affected the precision of the observed associations. In addition, the associations were based on unadjusted bivariate analyses; therefore, potential confounding cannot be excluded. Despite these limitations, the study provides valuable insights into awareness, perception, utilization, and challenges related to PM-JAY in an understudied population.

## Conclusions

The present community-based cross-sectional study conducted among peri-urban residents of Kanpur found that awareness of AB-PMJAY was moderate, with nearly three-fifths of the participants demonstrating good awareness of the scheme. Awareness was higher for major provisions, such as ₹5 lakhs annual coverage and free-of-cost card availability, whereas knowledge regarding important benefits, such as post-discharge care, family floater provisions, and eligibility criteria, remained inadequate. Although enrolment among eligible participants was relatively high, the actual utilization of PM-JAY services was considerably lower, indicating a significant gap between enrolment and effective use of the scheme. In addition, a substantial proportion of beneficiaries reported OOP expenditures and delays in receiving healthcare services despite being covered under PM-JAY, suggesting persistent implementation-level challenges.

## Appendices

Questionnaire on awareness, perception, utilization, and challenges related to Ayushman Bharat-Pradhan Mantri Jan Arogya Yojana (AB-PMJAY)

Instructions: Please select the most appropriate response. All information collected will be kept confidential and used only for research purposes.

Section A: Sociodemographic and General Information

1. Age

☐ <30 years ☐ 30-45 years ☐ 46-60 years ☐ ≥61 years

2. Gender

☐ Male ☐ Female ☐ Other

3. Marital status

☐ Married ☐ Unmarried ☐ Other

4. Educational status

☐ Illiterate ☐ Primary education ☐ Middle school ☐ High school ☐ Intermediate ☐ Graduate/postgraduate ☐ Professional education and above

5. Occupation

☐ Unskilled worker ☐ Semiskilled worker ☐ Skilled worker ☐ Clerk/farmer/shopkeeper ☐ Semiprofessional ☐ Professional ☐ Unemployed

6. Type of family

☐ Nuclear ☐ Joint ☐ Other

7. Number of family members

☐ ____________________________

8. Socioeconomic status

According to the modified B.G. Prasad socioeconomic classification used in the study.

☐ Lower class ☐ Lower-middle class ☐ Middle class ☐ Upper-middle class ☐ Upper class

9. Religion

☐ Hindu ☐ Muslim ☐ Other

10. Caste

☐ General ☐ Other backward class (OBC) ☐ Scheduled caste (SC) ☐ Scheduled tribe (ST)

11. Do you have a ration card?

☐ Yes ☐ No

Section B: Healthcare-Seeking Characteristics

1. How easy is it for you to access healthcare services when required?

☐ Easy ☐ Moderate ☐ Difficult

2. Who usually makes decisions regarding seeking healthcare for you/your family?

☐ Self ☐ Family member ☐ Others (friends/relatives)

3. How often do you visit a healthcare professional?

☐ Every week ☐ Every month ☐ Every 6 months ☐ Once a year ☐ Very rarely

4. What is your preference for healthcare for outpatient (OPD) services?

☐ Government ☐ Private

5. What is your preference for healthcare for inpatient (IPD) services?

☐ Government ☐ Private

Section C: Knowledge/Awareness Regarding AB-PMJAY

1. Are you aware of any government health schemes/health insurance schemes?

☐ Rashtriya Swasthya Bima Yojana (RSBY) ☐ Pradhan Mantri Jan Arogya Yojana (PM-JAY) ☐ Other: __________________

2. How familiar are you with AB-PMJAY/Golden Card?

☐ Not at all familiar ☐ Somewhat familiar ☐ Very familiar

3. How did you first learn about AB-PMJAY/Golden Card?

☐ Television ☐ Newspaper ☐ Radio ☐ Social media ☐ Word of mouth ☐ Other: __________________

4. Who do you think is eligible for AB-PMJAY/Golden Card?

☐ Based on socioeconomic criteria ☐ Based on caste ☐ Everyone ☐ Don't know

5. Do you think you are eligible for AB-PMJAY/Golden Card?

☐ Yes ☐ No ☐ Don't know

6. Are you aware of healthcare facilities in your area that participate in AB-PMJAY?

☐ Yes ☐ No

7. If yes, please name the healthcare facility.

Response to be verified for correctness.

☐ Name: ______________________________ ☐ Type: ☐ Public institution    ☐ Private institution

8. Under PM-JAY, are benefits provided:

☐ For each family member separately ☐ For the family ☐ Don't know

9. Is a separate card required for each family member?

☐ Yes ☐ No ☐ Don't know

10. What is the maximum annual coverage amount under PM-JAY?

☐ ₹ up to 30,000 ☐ ₹ up to 1 lakh ☐ ₹ up to 3 lakhs ☐ ₹ up to 5 lakhs ☐ More than ₹5 lakhs ☐ Don't know

11. Which of the following services do you think are covered under AB-PMJAY?

Multiple responses permitted.

☐ Outpatient (OPD) services ☐ Inpatient (IPD) services ☐ Emergency services ☐ Diagnostic services ☐ Transportation expenses ☐ Don't know

12. Are you aware of the portability of the PM-JAY card, i.e., its use outside your residential area/state?

☐ Yes ☐ No ☐ Don't know

13. Are you aware that a new family member can be added/registered under PM-JAY, such as a newborn child or newly married woman?

☐ Yes ☐ No ☐ Don't know

14. Are you aware of the grievance redressal mechanism under PM-JAY?

☐ Yes ☐ No ☐ Don't know

15. Is any premium payment required from the beneficiary for PM-JAY?

☐ Yes ☐ No ☐ Don't know

16. Is a pre-health check-up necessary to become eligible for AB-PMJAY/Golden Card?

☐ Yes ☐ No ☐ Don't know

17. Are you aware that pre-existing illnesses are covered under PM-JAY?

☐ Yes ☐ No ☐ Don't know

18. Are you aware of post-discharge benefits under PM-JAY?

☐ Yes ☐ No ☐ Don't know

Section D: Perception/Attitude Regarding AB-PMJAY

1. Do you believe that AB-PMJAY/Golden Card has improved access to healthcare in India?

☐ Strongly disagree ☐ Disagree ☐ Neutral ☐ Agree ☐ Strongly agree

2. How would you rate the quality of healthcare services received under AB-PMJAY/Golden Card?

☐ Poor ☐ Fair ☐ Good ☐ Very good ☐ Excellent

3. To what extent do you think AB-PMJAY/Golden Card has reduced out-of-pocket healthcare expenditure for economically vulnerable patients?

☐ Not at all ☐ To a small extent ☐ To a moderate extent ☐ To a large extent ☐ Completely

4. How satisfied are you with the coverage and benefits offered by AB-PMJAY/Golden Card?

☐ Completely dissatisfied ☐ Dissatisfied ☐ Neutral ☐ Satisfied ☐ Completely satisfied ☐ Don't know

5. How likely are you to recommend AB-PMJAY/Golden Card to others who need healthcare support?

☐ Very unlikely ☐ Unlikely ☐ Neutral ☐ Likely ☐ Very likely

6. How confident are you in the transparency and fairness of the PM-JAY application and approval process?

☐ Not confident at all ☐ Somewhat confident ☐ Neutral ☐ Very confident

7. Do you think there is a need for greater awareness and outreach programs to inform potential beneficiaries about AB-PMJAY/Golden Card?

☐ Strongly disagree ☐ Disagree ☐ Neutral ☐ Agree ☐ Strongly agree

8. Are you satisfied with the range of healthcare providers (public and private) available under AB-PMJAY in your area?

☐ Very dissatisfied ☐ Dissatisfied ☐ Neutral ☐ Satisfied ☐ Very satisfied

Section E: Utilization and Challenges Regarding AB-PMJAY

1. Do you currently have an AB-PMJAY/Golden Card?

☐ Yes ☐ No

2. Have you or any of your family members personally used the AB-PMJAY/Golden Card to access healthcare services?

☐ Yes ☐ No

3. If no, what was the reason for not using the AB-PMJAY/Golden Card?

Multiple responses permitted.

☐ Problem in obtaining the Golden Card, including difficulty in matching personal details with identification documents ☐ Procrastination/postponement of treatment ☐ Unaware of the benefits ☐ Did not know how to avail the benefits ☐ Other: __________________

4. If yes, which benefits did you receive?

Multiple responses permitted.

☐ Hospitalization expenses ☐ Pre-hospitalization expenses ☐ Post-hospitalization expenses ☐ Diagnostic services ☐ Medicines ☐ Other: __________________

5. What was the approximate distance between your house and the empanelled healthcare center where PM-JAY treatment was received?

☐ <10 km ☐ 10 to <50 km ☐ 50 to <100 km ☐ ≥100 km

6. How would you rate the ease of the enrolment/application process for AB-PMJAY (obtaining the Golden Card)?

☐ Very difficult ☐ Difficult ☐ Neutral ☐ Easy ☐ Very easy

7. Have you experienced any delays in receiving healthcare services under AB-PMJAY, such as hospital admission, treatment, or discharge?

☐ Never ☐ Rarely ☐ Occasionally ☐ Frequently ☐ Always

8. Did you incur any out-of-pocket expenditure while receiving healthcare services under PM-JAY?

☐ Yes ☐ No

9. Have you made any formal complaints or sought assistance regarding your AB-PMJAY/Golden Card benefits?

☐ Yes ☐ No

10. If yes, how would you rate the responsiveness and effectiveness of the PM-JAY customer support/helpline in addressing your queries or problems?

☐ 1 = Not responsive ☐ 2 ☐ 3 ☐ 4 ☐ 5 = Very responsive

11. How many times have you used the benefits of AB-PMJAY/Golden Card?

☐ ____________________________

12. How many members of your family have used the benefits under AB-PMJAY/Golden Card?

☐ ____________________________

13. How many times have your treatment costs exceeded the coverage amount under AB-PMJAY/Golden Card?

☐ Never ☐ Once ☐ More than once

14. For which specific type of treatment did you or your family members use AB-PMJAY/Golden Card during the past year?

Multiple responses permitted.

☐ Medicine/Paediatrics ☐ Surgery ☐ Orthopaedics ☐ Emergency Services ☐ Obstetrics and Gynaecology ☐ Other: __________________

15. Did you face any problems with waiting times at empanelled healthcare centers while using PM-JAY benefits?

☐ Yes ☐ No

16. Did you face any challenges in obtaining the necessary documentation or paperwork for availing PM-JAY benefits?

☐ Yes ☐ No

17. During the past year, did you or any of your family members face difficulty in accessing specialized or advanced medical treatment that was not covered under the AB-PMJAY/Golden Card scheme?

☐ Yes ☐ No

Thank you for participating in the study.
